# Neurometabolic Signatures of Alexithymia and Visuospatial Abilities in Parkinson’s Disease: An Exploratory ^1^H-MRS Study of the Substantia Nigra and Globus Pallidus

**DOI:** 10.3390/jcm15114236

**Published:** 2026-05-30

**Authors:** Laura Culicetto, Giulia Marafioti, Lilla Bonanno, Rosa Morabito, Gianluca Elio Fallica, Chiara Sorbera, Giuseppe Di Lorenzo, Silvia Marino, Angelo Quartarone, Rosella Ciurleo

**Affiliations:** IRCCS Centro Neurolesi Bonino-Pulejo, S.S. 113, Via Palermo C. da Casazza, 98124 Messina, Italy; laura.culicetto@irccsme.it (L.C.);

**Keywords:** Parkinson’s disease, Proton Magnetic Resonance Spectroscopy, substantia nigra, globus pallidus, visuospatial abilities, alexithymia

## Abstract

**Background:** Parkinson’s Disease (PD) is a multisystem neurodegenerative disorder associated with cognitive and emotional disturbances, including visuospatial deficits and alexithymia, which may substantially affect quality of life (QoL). The metabolic underpinnings of non-motor and emotional features within deep basal ganglia nuclei remain poorly understood. This exploratory proof-of-concept study aimed to examine ^1^H-MRS-derived metabolite ratios in the substantia nigra (SN) and globus pallidus (GP) and to explore their preliminary associations with visuospatial-attentional abilities and alexithymia. **Methods:** Fifteen individuals with PD and 15 healthy controls (HCs) underwent Proton Magnetic Resonance Spectroscopy (^1^H-MRS) targeting the SN and GP bilaterally. Metabolite ratios were quantified with LCModel and analyzed as left, right, and hemisphere-averaged measures. PD participants completed a multidisciplinary assessment including motor severity, cognition, visuospatial abilities, mood and alexithymia. Multiple testing was controlled using false discovery rate (FDR). Given the between-group imbalance in age and education, exploratory covariate-adjusted sensitivity analyses were also performed. **Results:** PD participants were older, less educated, and showed lower global cognition than HCs, including significantly reduced MoCA scores (20.9 ± 6.6 vs. 28.7 ± 1.8; FDR-corrected *p* < 0.001). In uncorrected analyses, between-group metabolite comparisons showed lower myo-inositol (Ins) in the SN (*p* = 0.04) and higher glutamatergic signal in the right GP in PD relative to HCs (*p* = 0.03); however, these differences were not robust after adjustment for age, education and multiple testing. Within the PD group, an uncorrected right–left asymmetry was observed for pallidal Ins. Exploratory correlations suggested uncorrected associations between SN metabolites and alexithymia dimensions related to emotional awareness and verbalization, whereas GP metabolites were more frequently associated with selected visuospatial, attentional, language-related, and broader cognitive measures. None of these associations survived FDR correction. **Conclusions:** This exploratory proof-of-concept study provides preliminary feasibility data and effect-size estimates for future ^1^H-MRS investigations of basal ganglia metabolites in PD. Given the small sample size, lack of cognitive matching, age and education imbalance, and absence of correction-surviving associations, the findings should not be interpreted as evidence of PD-specific neurometabolic markers. Larger, prospectively matched, and adequately powered studies are needed.

## 1. Introduction

Parkinson’s Disease (PD) is a progressive neurodegenerative disorder traditionally defined by its cardinal motor symptoms, including bradykinesia, rigidity, resting tremor, and postural instability [1]. However, it is now widely recognized as a multisystem disorder in which non-motor symptoms (NMSs) are highly prevalent and often more disabling than motor deficits [2]. Cognitive impairment, mood disturbances, autonomic dysfunction, sleep disorders, and alterations in emotional processing frequently occur across disease stages and substantially affect quality of life (QoL) [3,4]. Recent evidence has also raised the possibility that systemic metabolic factors, such as hypoglycemic episodes and glycemic variability, may be clinically relevant in parkinsonian syndromes, potentially contributing to symptom variability and disease complexity [5].

From a neurobiological perspective, this clinical heterogeneity reflects neurodegeneration that extends beyond dopaminergic neurons of the substantia nigra pars compacta to involve widespread neurotransmitter systems and distributed brain networks. Cognitive dysfunction in PD has been consistently linked to disruption of fronto-striatal and posterior cortical circuits, with contributions from dopaminergic, cholinergic, and glutamatergic abnormalities [6,7]. Even in the absence of dementia, individuals with PD frequently exhibit deficits in executive functioning, attention, and visuospatial abilities, domains critically dependent on fronto-striatal network integrity [8].

Neuropsychiatric symptoms in PD, including depression and anxiety, have been associated with dysfunction in limbic–basal ganglia loops and monoaminergic brainstem projections [9]. Within this framework, increasing attention has been directed toward alterations in emotional processing, particularly alexithymia, which appears more frequent in PD than in the general population [10]. Alexithymia, characterized by difficulty in identifying and describing one’s own feelings and emotions [11], is thought to reflect disruption of fronto-insular-cingulate networks involved in interoception and emotional appraisal [12]. In PD, alexithymia has been associated with depressive symptoms, apathy, and cognitive dysfunction, and may also relate to executive and visuospatial deficits, suggesting involvement of broader cortico-subcortical networks beyond limbic circuits alone [13,14]. These observations position alexithymia as a clinically relevant manifestation of network dysfunction in PD, potentially reflecting the interaction between limbic, interoceptive, and executive control systems.

Together, these findings support network-based models of PD in which motor, cognitive, and affective manifestations arise from dysfunction within partially overlapping fronto-striato-limbic circuits. Within this framework, the basal ganglia serve as integrative hubs linking motor, cognitive, limbic, and autonomic domains through parallel, partially segregated cortico-subcortical loops [15]. These circuits are strongly modulated by dopaminergic transmission, and degeneration of dopamine-dependent signaling within basal ganglia–prefrontal and basal ganglia–limbic pathways may therefore contribute not only to motor impairment but also to deficits in executive control, visuospatial processing, and emotional awareness, including alexithymia [16].

Within this system, the substantia nigra (SN) and globus pallidus (GP) occupy pivotal positions in motor, associative, and limbic loops, contributing to the modulation of executive functions, motivational states, and emotional processing [17,18]. Dysfunction within these interconnected circuits has been implicated in cognitive slowing, affective symptoms, sleep disturbances, and autonomic dysregulation in PD, supporting the view that neurochemical and metabolic alterations in basal ganglia nuclei may underlie both motor and non-motor phenotypes.

Proton Magnetic Resonance Spectroscopy (^1^H-MRS) provides a non-invasive in vivo method to investigate brain neurochemistry and offers insight into neuronal integrity, membrane turnover, glial metabolism, and neurotransmitter cycling [19]. Key metabolites measurable with ^1^H-MRS include N-acetylaspartate (NAA), considered a marker of neuronal viability and mitochondrial function; choline-containing compounds (Cho), reflecting membrane turnover; glutamate and glutamine (Glx), involved in excitatory neurotransmission; and myo-inositol (Ins), commonly interpreted as a marker of glial metabolism and osmotic regulation and often associated with neuroinflammatory or gliotic processes [20].

^1^H-MRS studies in PD have reported region-specific alterations in these metabolites, particularly within the SN, striatum, and other basal ganglia structures. Reductions in NAA consistent with neuronal dysfunction, changes in Ins suggestive of glial involvement, and abnormalities in Glx indicating altered excitatory neurotransmission have all been described [21,22,23,24]. However, most of these investigations have focused on motor-related regions or global cognitive decline, whereas the neurochemical substrates of emotional and higher-order cognitive disturbances, especially alexithymia and visuospatial–executive deficits, at the level of deep basal ganglia nuclei remain largely unexplored.

The present study aimed to investigate the neurochemical substrates of alexithymia and selected visuospatial-attentional abilities in PD by quantifying metabolite concentrations in the SN and GP using ^1^H-MRS. These regions were selected because they constitute key nodes within motor, associative, and limbic fronto-striatal circuits and are critically involved in PD pathophysiology. By integrating spectroscopy with detailed cognitive and affective assessments, including multidimensional measures of alexithymia, we sought to determine whether specific neurometabolic patterns are associated with distinct clinical domains.

The present proof-of-concept study aimed to explore whether ^1^H-MRS-derived metabolite ratios from the SN and GP could be feasibly examined in relation to selected cognitive and affective measures in PD. Given the limited sample size and exploratory design, the study was not intended to test definitive disease-specific biomarkers, but rather to generate preliminary effect-size estimates and methodological information for future adequately powered investigations. We expected that exploratory analyses might reveal preliminary patterns of association between basal ganglia metabolite variability and selected clinical, cognitive, and affective measures. These analyses were considered hypothesis-generating.

## 2. Materials and Methods

### 2.1. Study Participants and Design

This exploratory study included fifteen people with PD recruited from the Movement Disorders outpatient clinic and/or the Functional Rehabilitation of the IRCCS Centro Neurolesi “Bonino-Pulejo” in Messina, Italy, between November 2023 and January 2025.

Inclusion criteria were as follows: (i) male or female aged between 55 and 75 years; (ii) having a diagnosis of idiopathic Parkinson’s disease according to the United Kingdom Parkinson’s Disease Society Brain Bank criteria; (iii) Hoehn and Yahr stage ranging from 2 to 4; and (iv) receiving stable dopaminergic treatment, including levodopa and/or dopamine agonists, for at least six weeks prior to study entry.

Exclusion criteria included: (i) the presence of atypical parkinsonism or other parkinsonian syndromes; (ii) severe postural instability; (iii) evident autonomic failure at disease onset; (iv) the presence of dementia or other neurological or psychiatric disorders; (v) sensory-motor deficits or visual impairments that could interfere with neuropsychological assessment; and (vi) absolute contraindications to magnetic resonance imaging. Although patients with dementia were excluded on clinical grounds, no formal PD-MCI diagnostic classification was applied; cognitive status was therefore described on the basis of the clinical and neuropsychological assessment performed in the study.

Additionally, no formal stratification into PD subtypes was performed (e.g., tremor-dominant versus postural instability/gait difficulty phenotype, cognitive subtype, or non-motor subtype), because the modest sample size did not allow reliable subgroup analyses. However, for descriptive purposes, the side of predominant motor symptoms was recorded in the PD group: 10 out of 15 patients presented predominantly right-sided motor symptoms, whereas 5 out of 15 presented predominantly left-sided motor symptoms.

Eligible patients were assessed individually in a single comprehensive session by a multidisciplinary team consisting of a clinical psychologist and a neurologist specialized in movement disorders. Assessments were performed in a practically defined dopaminergic “OFF” state, before dopaminergic medication intake and under clinical supervision. However, the exact time elapsed since the last dopaminergic dose, and a formal standardized verification of OFF status were not systematically recorded. Demographic and clinical information were collected, including age, sex and years of education. Additional data regarding disease duration and pharmacological treatment, such as Levodopa Equivalent Daily Dose (LEDD), were also recorded.

The control group consisted of 15 HCs with no history of neurological or psychiatric disorders and normal cognitive functioning, as indicated by Mini-Mental State Examination (MMSE) scores > 26 and Montreal Cognitive Assessment (MoCA) scores > 23. The study was approved by the Ethics Committee of the IRCCS Centro Neurolesi “Bonino-Pulejo” (approval No. 43/2023) on 3 May 2023 and was conducted in accordance with the principles of the Declaration of Helsinki. All participants provided written informed consent prior to participation, and confidentiality of personal data was ensured.

### 2.2. Clinical and Neuropsychological Assessment

All PD subjects underwent a standardized neurological evaluation. Disease severity was classified according to the Hoehn and Yahr (H & Y) staging system, and motor symptoms were assessed using the Unified Parkinson’s Disease Rating Scale, Part III (UPDRS-III). In addition, a comprehensive battery of neuropsychological and functional measures was administered to assess cognitive and affective performance.

#### 2.2.1. Cognitive Function

- The Montreal Cognitive Assessment (MoCA) is a brief cognitive screening instrument consisting of a 30-point test administered on a single A4 sheet, with an average administration time of approximately 10 min [25]. A total score of 26 or higher is generally considered within the normal range. The MoCA was employed to assess five core cognitive domains. The short-term memory recall (5 points) was evaluated through two learning trials involving five nouns, followed by a delayed free recall after approximately 5 min. Visuospatial abilities were assessed by asking participants to reproduce a three-dimensional cube (1 point). Executive functions were examined using several tasks, including an alternating task adapted from the Trail Making Test part B (1 point), a clock-drawing task (3 points), a phonemic fluency task (1 point), and two verbal abstraction tasks (2 points). Attention, concentration, and working memory were measured through multiple components, such as a sustained attention task requiring target detection via tapping (1 point), serial subtraction (3 points), and both forward and backward digit span tasks (1 point each). Language abilities were assessed using a three-item confrontation naming task involving low-frequency animals (lion, camel, and rhinoceros; 3 points) and the repetition of two syntactically complex sentences (2 points).
- The Mini-Mental State Examination (MMSE) is a brief cognitive screening tool with a maximum score of 30 points, widely used to assess global cognitive function. It evaluates multiple cognitive domains, including temporal and spatial orientation, registration of new information, attention and calculation, short-term memory recall, language abilities, and visuospatial constructive skills. Interpretation of MMSE scores may vary according to the patient’s age and educational level. In general, scores ranging from 24 to 30 are considered within the normal range, scores between 18 and 23 indicate mild cognitive impairment, and scores below 18 suggest significant cognitive decline, potentially consistent with dementia [26].

Mood and Psychological Symptoms:

- Hamilton Rating Scale for Depression (HAM-D or HRS-D): A clinician-rated scale used to assess the severity of depressive symptoms. Scores range from 0 to 52, with a cut-off of ≥17 indicating clinically significant depression [26].
- Hamilton Rating Scale for Anxiety (HAM-A or HRS-A): A clinician-rated tool assessing the intensity of anxiety symptoms. The scale ranges from 0 to 56, with scores ≥ 18 reflecting clinically significant anxiety [27].

#### 2.2.2. Alexithymia Assessment

Given the multidimensional nature of alexithymia and the ongoing debate regarding its trait-like versus state-dependent components, we adopted a dual-measurement approach to obtain a more comprehensive assessment of emotional processing deficits. The 20-item Toronto Alexithymia Scale (TAS-20) is based on the original conceptualization of alexithymia as a relatively stable personality trait, characterized by difficulties in identifying and describing feelings, as well as a tendency toward externally oriented thinking [27,28]. In contrast, the Perth Alexithymia Questionnaire (PAQ) is grounded in the Attention–Appraisal Model [29], which conceptualizes alexithymia as a multidimensional construct reflecting impairments in emotional attention and appraisal processes. The PAQ was specifically developed to address limitations of earlier measures by differentiating between difficulties related to positive versus negative affect and between emotional awareness and emotional expression [30]. This dual-assessment approach allowed us to capture both trait-like and potentially state-sensitive aspects of alexithymia.

- The Toronto Alexithymia Scale (TAS-20) is a widely used and well-validated instrument for the assessment of alexithymia. It comprises three subscales: Difficulty Identifying Feelings (DIF; seven items), Difficulty Describing Feelings (DDF; five items), and Externally Oriented Thinking (EOT; eight items). Responses are provided on a five-point Likert scale ranging from 1 (strongly disagree) to 5 (strongly agree). According to established cut-off scores [27,28], total scores ≥ 61 indicate alexithymia, scores between 51 and 60 indicate borderline alexithymia, and scores ≤ 50 indicate non-alexithymia. In the present study, we used the Italian version of the TAS-20, validated by Bressi et al. (1996) [31], which demonstrates satisfactory internal consistency (Cronbach’s α = 0.75).
- The Perth Alexithymia Questionnaire (PAQ) [30] is a 24-item self-report measure designed to assess alexithymia across positive and negative emotional domains. It yields five subscales: Difficulty Identifying Negative Feelings (N-DIF), Difficulty Identifying Positive Feelings (P-DIF), Difficulty Describing Negative Feelings (N-DDF), Difficulty Describing Positive Feelings (P-DDF), and General Externally Oriented Thinking (G-EOT). These subscales can be summed to derive several composite indices, including a total alexithymia score. Items are rated on a seven-point Likert scale ranging from 1 (strongly disagree) to 7 (strongly agree), with higher scores reflecting greater levels of alexithymia.

#### 2.2.3. Visuospatial and Attentional Abilities

Visuospatial and attentional abilities were assessed using the six conventional subtests of the Behavioral Inattention Test–Conventional (BIT-C; Wilson, 1987 [32]).

- The Line Cancellation task consists of 40 short lines randomly distributed on an A4 landscape-oriented sheet, with 18 target lines presented on each side of the page. Four centrally located lines serve as practice items, are marked by the examiner, and are not included in scoring. The maximum score is 36, with a cutoff score of 34.
- The Letter Cancellation task comprises 40 target letters (E and R) embedded among 130 non-target letters on an A4 landscape-oriented sheet. The letters are arranged in five rows of 34 items each. Two additional target letters positioned below the stimulus rows are provided as practice examples and are not scored. The maximum score for this task is 40, with a cutoff score of 32.
- Figure and shape copying are assessed using two separate tasks. The figure-copying task includes three simple figures (a star, a cube, and a daisy) presented on the left side of an A4 portrait-oriented sheet. Participants are instructed to copy each figure into corresponding boxes located on the right side of the sheet. The shape-copying task consists of three geometric shapes displayed on an A4 landscape-oriented sheet, which participants are required to reproduce on a separate blank sheet. In both tasks, scoring is based on the completeness of the drawings, defined as the absence of omissions of major components. The maximum score is 4, with a cutoff score of 3.
- The Line Bisection task includes three horizontal lines (20.4 cm in length) arranged in a staircase configuration on an A4 landscape-oriented sheet. Participants are instructed to estimate and mark the midpoint of each line. Scoring is based on the magnitude of deviation from the true center of each line, yielding a maximum score of 9 and a cutoff score of 7.

In addition to the BIT-C, visuoconstructive and executive abilities were further assessed using the Clock Drawing Test (CDT). In CDT, participants were presented with an A4 sheet displaying a pre-drawn circle and were instructed to complete the clock face by inserting the numbers and drawing the hands to indicate the time “ten past eleven”. Performance was scored on a 5-point scale ranging from 0 to 5, with lower scores reflecting greater impairment in visuoconstructive and executive abilities [33].

### 2.3. ^1^H-MRS Acquisition and Analysis

All subjects were examined by using an MRI protocol which included combined conventional MRI and ^1^H-MRS examinations of the brain. The MRI acquisition was performed by 3T whole-body MRI equipment (Achieva, Philips Medical System, Best, The Netherlands), using a 32-element phased array sensitivity-encoding (SENSE) head coil.

The MR system was equipped with gradients, achieving a maximum slew rate of 200 mT/m/ms and maximum strength of 80 mT/m. The 3T imaging protocol included 3D T1-weighted Fast Field Echo (FFE), 3D FLAIR, two-dimensional coronal T2-weighted Fast Spin Echo (FSE). Three-dimensional-T1-weighted FFE images were acquired with the following parameters: Repetition Time (TR) 8.2 ms; Echo Time (TE) 3.7 ms; section thickness 1 mm; number of signals averaged 1 and reconstruction matrix 512 × 512. 3D-FLAIR images were acquired with TR 12,000 ms, TE 140 ms and T2-weighted. FSE images were acquired with TR 4100 ms, TE 100 ms, a section of thickness 2–3 mm, number of signals averaged to 1 and a reconstruction matrix of 512 × 512. The MR images were used to select intracranial volumes of interest (VOIs) for spectroscopy VOIs were placed bilaterally in the SN and GP using standardized anatomical landmarks on axial images.

Single-voxel ^1^H-MRS data were acquired using a point-resolved spectroscopy (PRESS) sequence (TE = 35 ms, TR = 2000 ms, 128 averages). The VOI of single-voxel MRS sections was 1.5 cm^3^. Corresponding unsuppressed water spectra with equal TE and TR were additionally acquired. Before data collection, the automatic shim provided by the manufacturer was carried out to optimize field homogeneity.

Metabolite resonance intensities were analysed using the LCModel/LCMgui software package (Version 6.3; Steven Provencher, Oakville, ON, Canada). Neurometabolite concentrations were estimated by fitting each spectrum to a linear combination of “basic spectra” of each neurometabolite, provided by LCModel software for a 3T PRESS acquisition with a TE = 35 ms.

Gaussian-fitted peak areas were quantified relative to a baseline calculated from a moving average of noise regions in the spectra. The primary neurometabolites identified included NAA at 2.0 ppm, Cr at 3.0 ppm, Cho at 3.2 ppm, Ins at 3.6 ppm, and Glx peak at 2.1–2.4 ppm. To ensure data quality, spectra with Cramer-Rao Lower Bounds exceeding 20% were excluded from the analysis. Neurometabolite levels were expressed as ratios to Cr (NAA/Cr, Cho/Cr, Ins/Cr, Glx/Cr) in the SN and GP. All spectra were acquired and analysed by an experienced evaluator blinded to participants’ PD status.

### 2.4. Statistical Analysis

Continuous variables were summarized as mean ± standard deviation (SD) or median and interquartile range (IQR), as appropriate, while categorical variables were reported as counts and percentages. Between-group comparisons were performed on demographic and global cognitive measures (age, years of education, MMSE, and MoCA) and on metabolite concentrations (NAA, Cho, Glx, and Ins) in the SN and GP (left and right hemispheres, as well as hemisphere-averaged unified measures). For continuous variables, distributional assumptions were assessed using the Shapiro–Wilk test and homogeneity of variances using Levene’s test. When both groups showed approximately normal distributions and equal variances, Student’s *t* test was used; when variances were unequal, Welch’s *t* test was applied. When at least one group deviated from normality, non-parametric comparisons were performed using the Mann–Whitney U test. Effect sizes for parametric tests were quantified as Hedges’ g, whereas for Mann–Whitney tests we computed rank-biserial correlation (δ), with the sign defined as PD minus HCs. Sex distribution between PD and HCc was compared using the χ^2^ test (or Fisher’s exact test in case of sparse contingency tables). Within-group hemispheric asymmetry was evaluated separately in PD and HCs by comparing right vs. left metabolite concentrations using paired analyses; the Shapiro–Wilk test was applied to the within-subject (right–left) differences to guide the choice between paired *t*-tests and Wilcoxon signed-rank tests. Effect sizes were quantified as Cohen’s d for paired *t*-tests and rank-biserial correlation for Wilcoxon tests. Within the PD group, correlation analyses were performed between unified metabolite measures and an extensive set of clinical, neuropsychological and non-motor symptom scales. TAS-20 and PAQ total scores and subscales were treated as separate exploratory variables in these analyses in order to capture complementary dimensions of alexithymia. No additional correction was applied specifically for conceptual overlap between alexithymia subscales, as multiple testing was controlled across the full family of PD-only metabolite-clinical correlations using the Benjamini–Hochberg procedure. Pearson correlation was used for continuous variables; Spearman’s rank correlation was applied for non-normally distributed. Point-biserial correlation was used for continuous-dichotomous associations. Given the between-group imbalance in age and education, we additionally performed an exploratory covariate-adjusted sensitivity analysis for PD–HCs comparisons using linear regression models with each metabolite as the dependent variable and group as the main predictor, adjusting for age and years of education. All tests were two-sided. To account for multiple comparisons, false discovery rate (FDR) correction using the Benjamini–Hochberg procedure was applied separately to families of tests (demographic comparisons, metabolite between-group comparisons, hemispheric within-group comparisons, and PD-only correlation analyses). For the PD-only correlation analyses, the FDR correction was applied across the full family of 480 metabolite-clinical correlations (8 unified metabolite measures × 60 clinical/neuropsychological variables). An FDR-corrected *p*-value < 0.05 was considered statistically significant. All analyses were performed in R (version 4.2.2).

## 3. Results

### 3.1. Descriptive Characteristics

The PD sample comprised 15 patients (10 males, 5 females) with a mean age of 64.3 ± 8.3 years and variable disease severity, as reflected by a mean Hoehn & Yahr stage 2.3 ± 0.6 and a mean UPDRS-III 32.7 ± 12.5. Global cognitive performance was reduced, with mean MMSE and MoCA scores of 24.7 ± 6.0 and 20.9 ± 6.6, respectively. Although participants with dementia were excluded, the PD group showed lower global cognitive performance than HCs. Importantly, PD and HC participants were not matched for global cognitive status, and no formal classification into cognitively normal PD versus PD-MCI was performed. Therefore, the present design does not allow us to determine whether the observed patterns are specific to PD or partly reflect differences in global cognitive performance. Patients also showed relevant anxiety and depressive symptoms (mean HARS 17.0 ± 6.7; mean HDRS 14.5 ± 4.9) (Table 1).

The HCs group (n = 15) was younger and more educated (mean age 55.7 ± 3.7 years; mean education 14.5 ± 4.0 years), with preserved global cognition (mean MMSE 29.5 ± 1.0; mean MoCA 28.7 ± 1.8) (Table 1).

### 3.2. Intra-Group Comparisons

Within-group hemispheric analyses (right vs. left) showed an uncorrected right–left difference only for Ins in the GP in the PD group, with lower values in the left hemisphere (mean_left = 0.52 ± 0.21 vs. mean_right = 0.69 ± 0.28; mean difference = 0.17). This effect was significant at the uncorrected level (paired *t*-test: t = 2.60, *p* = 0.021; Cohen’s d = 0.67), but did not survive FDR correction (p_FDR = 0.17). All other hemispheric comparisons in PD were not significant after correction (Table 2). In the HCs group, no hemispheric differences were observed for any metabolite (Table 2).

### 3.3. Between-Group Comparisons

For the metabolite comparisons (Table 2), PD subjects showed an uncorrected lower Ins concentrations in the SN (unified) (δ = −1.51; *p* = 0.04) and in the left SN (δ = −1.61; *p* = 0.01). In uncorrected analyses, PD subjects showed higher Glx levels in the right GP compared with HCs (δ = 0.61, *p* = 0.03). For Mann–Whitney comparisons, the rank-biserial correlation (δ) was oriented such that positive values indicate higher metabolite levels in PD compared with HC whereas negative values indicate lower levels in PD. However, none of these differences survived FDR correction and the subsequent covariate-adjusted sensitivity analyses did not support robust between-group metabolite differences after controlling for age and education.

### 3.4. Correlations Between Metabolites and Clinical Measures in PD

Given the limited sample size and the large number of comparisons, we a priori prioritized hemisphere-averaged (unified) metabolite measures for correlation analyses to improve stability and reduce the multiplicity burden. Hemisphere-specific correlations were considered exploratory and were performed only as sensitivity analyses for metabolites showing significant right–left asymmetry.

**Figure 1:**
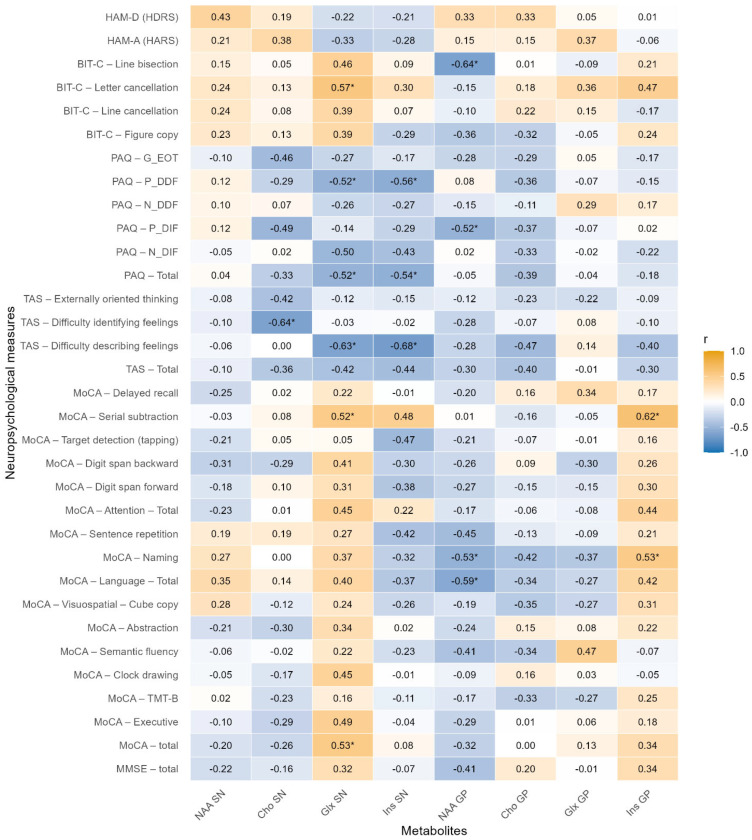
Correlation matrix between unified ^1^H-MRS metabolite concentrations and neuropsychological measures in the PD patients. Each cell reports the correlation coefficient with asterisks denoting uncorrected * *p* < 0.05. Color scale encodes direction and magnitude (negative = blue, positive = orange). Legend: Choline-containing compounds = Cho; Globus Pallidus = GP; glutamate and glutamine = Glx; Myo-inositol = Ins; N-acetylaspartate = NAA; Substantia Nigra = SN; Hamilton Depression Rating Scale = HAM-D (HDRS); Hamilton Anxiety Rating Scale = HAM-A (HARS); Behavioral Inattention Test—Conventional = BIT-C; Toronto Alexithymia Scale = TAS; Externally Oriented Thinking = EOT; Difficulty Identifying Feelings = DIF; Difficulty Describing Feelings = DDF; Montreal Cognitive Assessment = MoCA; Mini-Mental State Examination = MMSE; Trail Making Test—Part B = TMT-B.

Within the PD group, exploratory correlation analyses identified several uncorrected associations between metabolite concentrations and cognitive, neuropsychological, and affective measures (Figure 1). At the significant at the uncorrected level, Cho_SN was negatively associated with TAS-DDF (r = −0.64, *p* = 0.01). NAA_GP was negatively associated with naming performance (r = −0.53, *p* = 0.04), and Glx_SN was negatively associated with TAS-DIS (r = −0.63, *p* = 0.01). Spearman correlations further suggested that higher Ins_SN levels were associated with lower TAS-DIS (ρ = −0.68, *p* = 0.006), lower P-DDF (ρ = −0.56, *p* = 0.03), and lower PAQ total scores (ρ = −0.54, *p* = 0.04). NAA_GP was also negatively correlated with line bisection performance (ρ = −0.64, *p* = 0.01), total language score (ρ = −0.59, *p* = 0.02), and naming (ρ = −0.53, *p* = 0.04). Glx_SN showed a positive association with letter cancellation performance (ρ = 0.57, *p* = 0.03) and MoCA scores (ρ = 0.53, *p* = 0.04). In addition, Ins_GP was positively correlated with serial subtraction performance (ρ = 0.62, *p* = 0.01) and naming (ρ = 0.53, *p* = 0.04). However, none of these correlations survived FDR correction. In the hemisphere-specific sensitivity analysis restricted to the metabolite showing right–left asymmetry (Ins_GP), no correlation with cognitive and neuropsychological measures remained significant after FDR correction. However, left-sided Ins_GP was positively correlated with MoCA (ρ = 0.55, *p* = 0.03), letter cancellation (ρ = 0.61, *p* = 0.01), naming (ρ = 0.54, *p* = 0.03), and the executive domain score (ρ = 0.55, *p* = 0.04).

### 3.5. Covariate-Adjusted Sensitivity Analysis

Given the significant between-group imbalance in age and education, we conducted a covariate-adjusted sensitivity analysis to better contextualize the unadjusted PD-HC metabolite comparisons. Across metabolites, the adjusted PD–HCs group effect was generally small and not statistically significant. The largest adjusted group effect was observed for Glx_GP right, showing higher values in PD compared with HCs (β = 0.77, SE = 0.38, t = 2.01, *p* = 0.05; 95% CI −0.02 to 1.55), although this association did not survive FDR correction (p_FDR = 0.98). All remaining adjusted group effects were non-significant (all *p* > 0.05; all p_FDR = 0.98), indicating that the previously observed unadjusted between-group trends were not robust after accounting for age and education (Table S1).

## 4. Discussion

The present study should be interpreted as an exploratory proof-of-concept investigation. Its main contribution is not the identification of definitive PD-specific neurometabolic markers, but the demonstration of feasibility, the description of effect sizes, and the identification of methodological issues that should guide future adequately powered studies. Overall, no between-group metabolite difference or metabolite–clinical association survived correction for multiple comparisons, and unadjusted trends were further weakened after adjustment for age and education.

Accordingly, the observed uncorrected associations should not be interpreted as evidence of disease-specific mechanisms. Rather, they may help generate hypotheses regarding which brain regions, metabolites, and clinical variables may be prioritized in future studies.

In patients with PD, metabolic alterations have been reported in brain regions associated with NMSs, including the cerebral cortex, prefrontal lobe, hippocampus, and posterior cortical areas, as well as in the SN, GP, thalamus, and putamen [34,35,36,37,38]. These findings suggest that ^1^H-MRS can provide insights into the neurochemical substrates underlying non-motor features. Importantly, these neurometabolic changes, which have been shown to correlate with NMSs [34,38], precede visible tissue loss but are not detectable with structural MRI, which primarily identifies macroscopic atrophy or structural abnormalities, or with functional MRI (fMRI), which can detect altered connectivity patterns in networks subserving cognition and emotion but does not directly assess the underlying neurochemical environment. However, when compared with positron emission tomography (PET) and single-photon emission computed tomography (SPECT), ^1^H-MRS demonstrates important limitations. PET imaging offers high sensitivity and specificity for neurotransmitter dysfunction that is directly relevant to non-motor symptoms such as depression and cognitive impairment [39], while ^1^H-MRS does not directly measure dopaminergic neurotransmission, which remains central to the pathophysiology and clinical diagnosis of PD. In fact, dopamine transporter imaging with SPECT or fluorodopa PET retains superior sensitivity and specificity for detecting nigrostriatal dysfunction, particularly in early-stage disease. Nevertheless, although its clinical applicability is limited by several technical factors, including complex data processing, limited standardization, susceptibility to artifacts, and difficulties in metabolite quantification, ^1^H-MRS offers several advantages in the evaluation of NMSs in PD. Its non-invasive nature and absence of ionizing radiation make it well suited for longitudinal studies, enabling repeated assessments of disease progression and therapeutic response [40]. This is particularly valuable for tracking the evolution of cognitive impairment and neuropsychiatric symptoms over time.

Although unadjusted between-group comparisons suggested nominal differences in SN Ins and right GP Glx, these trends were not confirmed after adjustment for age and education. Within the PD group, SN metabolites were more frequently associated with alexithymia-related dimensions, whereas GP metabolites were more often associated with visuospatial, attentional, language-related, and broader cognitive measures. Overall, the FDR-corrected results were essentially null for both metabolite-clinical correlations and between-group metabolite comparisons; therefore, all nominal associations should be interpreted cautiously and considered hypothesis-generating only. In addition, a nominal right–left asymmetry was observed for pallidal Ins in the PD group. Taken together, these exploratory results may help identify candidate regions, metabolic and clinical domains that could be prioritized in future adequately powered studies.

From a clinical perspective, the PD group showed clinical features broadly compatible with the multisystem nature of PD, including lower global cognitive performance than HCs and affective burden. This interpretation is consistent with the broader literature showing that PD involves not only motor symptoms, but also emotional and cognitive disturbances supported by dysfunction in striato-thalamo-cortical and mesolimbic networks [41].

With respect to between-group metabolite differences, unadjusted analyses suggested nominally lower Ins levels in the SN and nominally higher Glx levels in the right GP in PD relative to HCs. However, these trends were attenuated and no longer supported in the covariate-adjusted analyses controlling for age and education, indicating that they should not be interpreted as robust group differences. Although preliminary, this pattern may be of potential pathophysiological interest. Ins has often been interpreted in relation to glial metabolism and osmotic regulation [42,43], whereas Glx is commonly regarded as an index of glutamatergic–glutaminergic metabolism. Within this framework, the observed pattern may tentatively reflect altered metabolic balance in deep basal ganglia nuclei that are central to PD pathophysiology. This interpretation is broadly consistent with previous literature implicating neuroinflammatory mechanisms in the nigrostriatal system [44,45] and altered excitatory neurotransmission within basal ganglia circuits following dopaminergic depletion [46]. However, because these effects were attenuated after adjustment for age and education, they should be interpreted with caution.

More broadly, these dopamine-modulated circuits should be considered within the progressive course of neurodegeneration in PD, in which pathology extends beyond the nigrostriatal system to affect associative, limbic, and cortico-subcortical networks, thereby contributing to the evolving cognitive, affective, and non-motor manifestations of the disease.

One of the most distinctive aspects of the present study concerns the nominal association between SN metabolites and alexithymia-related dimensions. Several nominal correlations involved TAS-20 and PAQ dimensions related to difficulty identifying and describing feelings, suggesting that metabolic variability in the SN may be associated with aspects of emotional awareness and emotional verbalization. This interpretation is plausible in light of previous work indicating that alexithymia is relatively frequent in PD and is associated with neuropsychiatric and cognitive features. In particular, Assogna et al. (2016) highlighted the relevance of alexithymia in PD, while Costa et al. (2007) reported that alexithymic PD patients may show poorer performance on tasks involving visuospatial abilities [14,47].

These findings are also biologically plausible in light of the broader functional role of the SN. Although traditionally viewed as a core motor structure, the SN also contributes to associative, motivational, and limbic processing through its connections with basal ganglia and cortico-limbic circuits [48,49]. Degeneration of dopaminergic and noradrenergic pathways within these networks has been linked to depression, anxiety, and broader affective dysregulation in PD [9]. Within this neurobiological context, our findings are consistent with the possibility that alexithymia-related traits in PD may be associated, at least in part, with subcortical metabolic variability, linked to neuro-glia alterations and glutamatergic dysregulation, particularly within the SN.

A partially and preliminary distinct pattern emerged for cognitive performance. Compared with SN metabolites, GP metabolites were more often associated with line bisection, letter cancellation, naming, serial subtraction, and global cognitive measures such as the MoCA. This distribution of associations may be broadly compatible with the recognized role of pallidal structures in broader cognitive and attentional processes [50]; however, given the absence of a dedicated executive test battery, these findings should not be interpreted as providing specific evidence of executive or attentional network dysfunction. Notably, alexithymia scores were not directly associated with visuospatial or broader cognitive performance at the behavioral level. However, the parallel association of alexithymia-related and cognitive measures with distinct subcortical metabolic markers may be compatible with the possibility that emotional awareness deficits and higher-order cognitive dysfunction in PD reflect partially dissociable, yet interconnected, manifestations of broader cortico-subcortical network disruption. This interpretation is in line with previous evidence that alexithymia in PD can co-occur with visuospatial and executive difficulties, pointing to a broader network vulnerability beyond purely limbic circuits [47].

Within the PD group, we also observed a nominal right–left asymmetry in pallidal Ins, with lower values in the left hemisphere. Although the biological significance of this finding remains uncertain, metabolic asymmetries may plausibly relate to the well-known lateralization of nigrostriatal degeneration in PD [51] and warrant further investigation in larger samples stratified by side of onset and motor phenotype. In this respect, hemispheric metabolic variability may represent an additional dimension of clinical heterogeneity that remains insufficiently explored in current spectroscopy studies.

Overall, the present findings may be compatible with a network-based interpretation of non-motor manifestations in PD, although the cognitive assessment used here does not allow precise inferences regarding specific executive or attentional networks. Rather than reflecting a single undifferentiated process, emotional awareness and cognitive performance may relate to partially distinct aspects of basal ganglia neurometabolism. In the present sample, SN-related variability appeared more closely linked to alexithymia-related dimensions, whereas GP-related variability was more frequently associated with visuospatial-attentional and broader cognitive measures. Although preliminary, this pattern is conceptually relevant because it may suggest that different basal ganglia nodes are differentially associated with emotional and cognitive features in PD.

More broadly, the present study highlights the value of combining ^1^H-MRS with multidimensional neuropsychological and affective assessment. Most spectroscopy studies in PD have focused primarily on motor symptoms or global cognitive decline, whereas emotional processing disturbances such as alexithymia have received comparatively less attention. By integrating metabolite profiles from deep gray matter nuclei with both TAS-20 and PAQ measures, the present work highlights emotional awareness as a potentially meaningful dimension for understanding disease heterogeneity.

### 4.1. Limitations

The modest sample size limited statistical power and increased the risk of both type I and type II errors, particularly given the large number of metabolites and clinical variables examined. In particular, the small sample size relative to the number of metabolites, bilateral regional measures and exploratory correlation analyses may have reduced statistical power and limited the stability of the observed associations. Despite attempts to reduce multiplicity by prioritizing hemisphere-averaged measures, none of the observed correlations survived FDR correction; therefore, all metabolite–clinical associations should be regarded as exploratory and hypothesis-generating. No a priori power analysis was performed, as the study was designed as an exploratory investigation and the sample size was determined by feasibility and participant availability during the recruitment period; therefore, the findings should be interpreted cautiously and primarily as hypothesis-generating. The cross-sectional design precludes conclusions about directionality or causality between neurometabolic alterations and emotional or cognitive features. Another methodological limitation concerns the assessment of patients in a practically defined dopaminergic OFF state, which was not operationalized using a fully standardized post-dose interval or a formal structured verification procedure for all participants. Therefore, some variability in motor state at the time of assessment cannot be entirely excluded. In addition, although patients were receiving stable dopaminergic treatment and LEDD was recorded, chronic medication exposure and treatment history may still have influenced both neuropsychological performance and ^1^H-MRS measures. Since LEDD was not incorporated as a formal covariate in the analyses, medication-related confounding cannot be ruled out. Although mood symptoms were assessed using HARS and HDRS, these variables were not included as covariates in the metabolite-alexithymia analyses; therefore, the potential overlap between alexithymia, depression, and anxiety cannot be excluded and should be considered when interpreting these exploratory associations. In addition, ^1^H-MRS provides indirect markers of neurochemical status and cannot specify the precise cellular or neurotransmitter mechanisms underlying the observed associations. An additional limitation is the significant demographic imbalance between groups (age and education). To partially address this issue, we performed covariate-adjusted sensitivity analyses (metabolite ~ group + age + education), which did not reveal any robust PD–HCs metabolite differences after correction for multiple testing (Table S1), further supporting the preliminary nature of the unadjusted between-group trends. A related issue is that alexithymia measures (TAS-20 and PAQ) were administered only to the PD group and not to HCs. As a result, we could not directly evaluate between-group differences in alexithymia, and the specificity of the observed alexithymia-related associations to PD should be interpreted cautiously. A further limitation is that no formal PD-MCI diagnostic classification was applied. Thus, although dementia was clinically excluded, cognitive impairment within the PD group was characterized only through screening and neuropsychological measures. In addition, the neuropsychological characterization of cognitive domains was limited by the absence of a dedicated and comprehensive executive test battery. Executive abilities, as well as other cognitive domains, were inferred largely from screening-derived subcomponents and task-specific visuospatial or attentional measures. This approach may have reduced sensitivity to subtle fronto-striatal deficits and limited the specificity of metabolite–cognition associations. Accordingly, interpretations involving specific executive or attentional network dysfunction should be considered preliminary and indirect. In addition, visuospatial functioning was assessed through BIT-C subtests and the CDT, which provide clinically informative but not comprehensive coverage of the visuospatial domain. Therefore, the present findings should be interpreted as referring to selected visuospatial-attentional and visuoconstructive measures rather than to visuospatial abilities more broadly.

Other limitations relate to certain technical aspects of ^1^H-MRS. Although Cr-referenced ratios are widely employed in MRS studies due to their robustness and favorable signal-to-noise properties, Cr levels may not be entirely stable in PD, particularly in regions such as the SN and GP. This introduces a potential source of bias in the interpretation of metabolite ratios. While unsuppressed water spectra were acquired, these were primarily used for spectral scaling and quality control rather than full absolute quantification. Water-referenced approaches, although potentially more accurate, require additional corrections for relaxation times (T1 and T2) and tissue composition, which are challenging to implement reliably in small brainstem structures due to partial volume effects and anatomical variability [52,53]. Therefore, Cr-referenced ratios were considered a pragmatic and robust approach for group comparisons in this context. Findings should be interpreted with caution and warrant confirmation with future studies focused on employing fully quantitative approaches.

Another limitation is that no tissue composition correction (grey matter, white matter, and cerebrospinal fluid fractions) was performed. Consequently, metabolite estimates may be influenced by partial volume effects. This is particularly relevant for small structures such as the SN and GP, where accurate segmentation is challenging due to their limited size, anatomical variability, and susceptibility-related distortions, which may reduce the reliability of tissue fraction estimates.

Finally, although voxel placement followed standardized anatomical criteria and a predefined protocol, inter-rater reliability was not formally assessed. As such, some degree of operator-dependent variability in voxel positioning cannot be excluded.

### 4.2. Future Directions

Future studies should be prospectively designed to reduce inter- and intra-subject variability. In particular, PD and HC groups should be matched for age, education, sex, handedness, and global cognitive status. Formal MDS PD-MCI criteria should be applied to distinguish cognitively normal PD from PD-MCI, and additional disease-control groups, such as non-PD MCI, Alzheimer’s disease, or vascular cognitive impairment, may help clarify the specificity of MRS findings. Medication state should be standardized using a predefined OFF interval, and detailed medication history should be recorded, including dopaminergic and non-dopaminergic drugs potentially affecting neuropsychological or MRS measures. Future protocols should also systematically record handedness, side of motor onset, predominant motor side, and motor phenotype. Finally, integration with dopaminergic imaging, such as DAT-SPECT/DATSCAN, may help determine whether asymmetric MRS signals relate to asymmetric nigrostriatal dopaminergic degeneration.

## 5. Conclusions

In conclusion, this exploratory proof-of-concept study provides preliminary feasibility data and effect-size estimates for future ^1^H-MRS studies of basal ganglia metabolites in PD. No between-group metabolite difference or metabolite–clinical association survived correction for multiple comparisons, and unadjusted trends were not robust after adjustment for demographic variables. Given the small sample size, lack of cognitive matching, age and education imbalance, and absence of formal PD-MCI classification, no firm conclusions can be drawn regarding PD-specific neurometabolic correlates of alexithymia or visuospatial performance. Future adequately powered studies should use prospectively matched samples, formal cognitive classification, detailed medication characterization, handedness and lateralization measures, and multimodal dopaminergic imaging to clarify whether basal ganglia metabolite variability contributes to non-motor heterogeneity in PD.

## Figures and Tables

**Table 1:** Demographic, cognitive characteristics in patients with PD and HC.

	PD	HC	Effect Size	*p*-Value
*Demographics*/*Clinical*				
Age	64.27 ± 8.33	55.73 ± 3.73	g = 1.29	0.002 *^¥^
Gender				
Male	10	8	φ = 0.14	0.46 ^χ^
Female	5	7		
Education	9.8 ± 4.65	14.53 ± 4.00	δ = −1.06	0.01 *^α^
Hoehn & Yahr	2.3 ± 0.6	-		
LEDD	707.67 ± 344.37	-		
MMSE	24.71 ± 6.00	29.48 ± 1.01	δ = −1.08	0.002 *^α^
MoCA	20.93 ± 6.62	28.67 ± 1.76	δ = −1.55	<0.001 *^α^
UPDRS-III	32.7 ± 12.5	-		
HARS	17.0 ± 6.7	-		
HDRS	14.5 ± 4.9	-		
TAS-20	53.87 ± 11.31	-		
PAQ	95.73 ± 35.29	-		

Group differences were tested using Welch’s *t*-test (^¥^), Mann–Whitney test (^α^) or χ^2^ test (^χ^) as appropriate. Effect sizes are reported as Hedges’ g for parametric comparisons and rank-biserial correlation (δ) for non-parametric comparisons. * *p* < 0.05. Legend: H&Y = Hoehnand Yahr scale; LEDD = L-dopa equivalent daily dose; MMSE = Mini Mental State Examination; MoCA = Montreal Cognitive Assessment; HARS = Hamilton Anxiety Rating Scale; HDRS = Hamilton Depression Rating Scale; UPDRS-III = Unified Parkinson’s Disease Rating Scale—Part III; TAS-20 = Toronto Alexithymia Scale; PAQ = Perth Alexithymia Questionnaire.

**Table 2:** Between and within-group hemispheric analyses of metabolite concentrations in PD patients and HCs.

	PD	HCs	Effect Size	*p*-Value
	Mean ± SD	Mean ± SD		
Cho GP	0.34 ± 0.13	0.30 ± 0.08	δ = 0.08	0.74 ^α^
Cho SN	0.35 ± 0.07	0.33 ± 0.07	g = 0.23	0.53 ^¥^
Ins GP	0.61 ± 0.21	0.67 ± 0.28	g = −0.26	0.48 ^¥^
Ins SN	0.62 ± 0.36	0.82 ± 0.35	δ = −0.44	0.04 *^α^
Glx GP	2.02 ± 0.60	1.77 ± 0.89	δ = 0.32	0.14 ^α^
Glx SN	2.08 ± 0.48	2.29 ± 1.11	g = −0.05	0.84 ^¥^
NAA GP	1.07 ± 0.15	1.07 ± 0.24	g = 0.0	0.99 ^¥^
NAA SN	1.32 ± 0.43	1.31 ± 0.29	δ = 0.03	0.92 ^α^
*Metabolites Variables for hemisphere*				
Cho GP Right	0.33 ± 0.18	0.29 ± 0.11	δ = −0.01	0.97 ^α^
Cho GP Left	0.36 ± 0.18	0.32 ± 0.09	δ = 0.03	0.90 ^α^
Effect size; *p*-value	r = −0.13; 0.67 ^µ^	d = −0.19; 0.48 ^ε^		
Cho SN Right	0.32 ± 0.09	0.35 ± 0.12	g = −0.21	0.55 ^¥^
Cho SN Left	0.37 ± 0.12	0.32 ± 0.10	g = 0.51	0.16 ^¥^
Effect size; *p*-value	r = −0.28; 0.35 ^µ^	d = 0.17; 0.51 ^ε^		
Ins GP Right	0.69 ± 0.28	0.71 ± 0.30	g = −0.04	0.90 ^¥^
Ins GP Left	0.52 ± 0.21	0.64 ± 0.38	g = −0.38	0.30 ^¥^
Effect size; *p*-value	d = 0.67; 0.02 ^ε^*	d = 0.18; 0.50 ^ε^		
Ins SN Right	0.60 ± 0.39	0.79 ± 0.42	g = −0.45	0.22 ^¥^
Ins SN Left	0.65 ± 0.47	0.85 ± 0.37	δ = −0.55	0.01 *^α^
Effect size; *p*-value	r = 0.05; 0.89 ^µ^	d = −0.17; 0.51 ^ε^		
Glx GP Right	2.07 ± 0.67	1.67 ± 0.90	δ = 0.46	0.03 *^α^
Glx GP Left	1.97 ± 0.71	1.87 ± 0.94	δ = 0.16	0.48 ^α^
Effect size; *p*-value	d = 0.14; 0.59 ^ε^	d = −0.40; 0.14 ^ε^		
Glx SN Right	2.16 ± 0.68	2.52 ± 1.69	δ = −0.02	0.93 ^α^
Glx SN Left	1.99 ± 0.50	2.07 ± 0.91	δ = 0.10	0.65 ^α^
Effect size; *p*-value	d = 0.24; 0.37 ^ε^	d = 0.29; 0.28 ^ε^		
NAA GP Right	1.03 ± 0.23	1.06 ± 0.36	g = −0.09	0.80 ^¥^
NAA GP Left	1.10 ± 0.29	1.07 ± 0.30	g = 0.09	0.79 ^¥^
Effect size; *p*-value	d = −0.17; 0.52 ^ε^	d = −0.03; 0.90 ^ε^		
NAA SN Right	1.40 ± 0.42	1.28 ± 0.48	g = 0.26	0.46 ^¥^
NAA SN Left	1.24 ± 0.52	1.34 ± 0.56	g = −0.17	0.63 ^¥^
Effect size; *p*-value	d = 0.41; 0.13 ^ε^	d = −0.07; 0.79 ^ε^		

Legend: Choline-containing compounds = Cho; Globus Pallidus = GP; glutamate and glutamine = Glx; HCs = Healthy Controls; Myo-inositol = Ins; N-acetylaspartate = NAA; Parkinson’s Disease = PD; Substantia Nigra = SN. * refers to statistical significance; Group differences were tested using Welch’s *t*-test (^¥^), Paired *t*-test (^ε^), Wilcoxon signed-rank (^µ^) and Mann–Whitney test (^α^) as appropriate. Effect sizes are reported as Hedges’ g for parametric comparisons and rank-biserial correlation (δ) for non-parametric comparisons. For Mann–Whitney tests, positive δ values indicate higher metabolite levels in PD than in HCs, whereas negative δ values indicate lower metabolite levels in PD.

## Data Availability

Data reported in this study are available from the corresponding author on reasonable request.

## Supplementary Materials

The following supporting information can be downloaded at: https://www.mdpi.com/article/10.3390/jcm15114236/s1, Table S1: Covariates-adjusted sensitivity analysis of metabolite concentrations in PD vs. HCs.
