# Supplementary material for: Neurometabolic Signatures of Alexithymia and Visuospatial Abilities in Parkinson’s Disease: An Exploratory 1H-MRS Study of the Substantia Nigra and Globus Pallidus
Source: J Clin Med. 2026 May 30;15(11):4236. doi: 10.3390/jcm15114236 (PMC13258415; doi:10.3390/jcm15114236)
Supplement: Supplementary file 1 [file jcm-15-04236-s001.zip › jcm-4264503-supplementary.pdf]

Table S1. Covariate-adjusted sensitivity analysis of metabolite concentrations in PD vs HCs.

Metabolite	β (PD vs HCs)	SE	t	p	95%CI	p_FDR
NAA SN	0.21	0.17	1.21	0.24	-0.15 to 0.56	0.98
NAA SN Right	0.26	0.22	1.18	0.25	-0.19 to 0.72	0.98
NAA SN Left	0.16	0.26	0.61	0.55	-0.37 to 0.69	0.98
Ins SN	−0.19	0.18	−1.07	0.29	-0.55 to 0.17	0.98
Ins SN Right	-0.05	0.20	-0.26	0.79	-0.46 to 0.35	0.98
Ins SN Left	−0.33	0.21	−1.57	0.13	-0.75 to 0.10	0.98
Cho SN	0.02	0.04	0.45	0.66	-0.06 to 0.09	0.98
Cho SN Right	0.004	0.05	0.07	0.94	-0.10 to 0.11	0.98
Cho SN Left	0.03	0.05	0.53	0.60	-0.11 to 0.14	0.98
Glx SN	0.05	0.42	0.12	0.90	-0.81 to 0.91	0.98
Glx SN Right	0.11	0.63	0.18	0.86	-1.18 to 1.40	0.98
Glx SN Left	−0.01	0.36	−0.02	0.98	-0.75 to 0.74	0.98
NAA GP	−0.03	0.09	−0.36	0.72	-0.22 to 0.16	0.98
NAA GP Right	−0.05	0.15	−0.33	0.74	-0.35 to 0.25	0.98
NAA GP Left	−0.02	0.14	−0.13	0.90	-0.30 to 0.26	0.98
Ins GP	−0.06	0.12	−0.45	0.66	-0.31 to 0.20	0.98
Ins GP Right	−0.08	0.14	−0.54	0.60	-0.37 to 0.22	0.98
Ins GP Left	−0.03	0.15	−0.23	0.82	-0.34 to 0.27	0.98
Cho GP	−0.04	0.05	−0.90	0.38	-0.14 to 0.05	0.98
Cho GP Right	−0.04	0.07	−0.59	0.56	-0.19 to 0.10	0.98
Cho GP Left	−0.04	0.06	−0.67	0.51	-0.17 to 0.09	0.98
Glx GP	0.36	0.38	0.95	0.35	-0.41 to 1.13	0.98
Glx GP Right	0.77	0.38	2.01	0.05	-0.02 to 1.55	0.98
Glx GP Left	−0.05	0.41	−0.12	0.91	-0.90 to 0.80	0.98

Legend: Choline-containing compounds = Cho; Globus Pallidus =GP; glutamate and glutamine = Glx; HCs = Healthy Controls; Ins = Myo-inositol; N-acetylaspartate = NAA; Parkinson's Disease = PD; Substantia Nigra = SN. Between-group differences were evaluated using linear regression models with metabolite concentration as the dependent variable and group (PD vs HCs) as the main predictor, adjusting for age and years of education. β coefficients represent the adjusted mean difference between PD and HCs. Standard errors (SE), t statistics, p-values, and 95% confidence intervals (CI) are reported. False discovery rate (FDR) correction using the Benjamini–Hochberg procedure was applied across all metabolite comparisons. None of the adjusted between-group comparisons remained significant after FDR correction.
